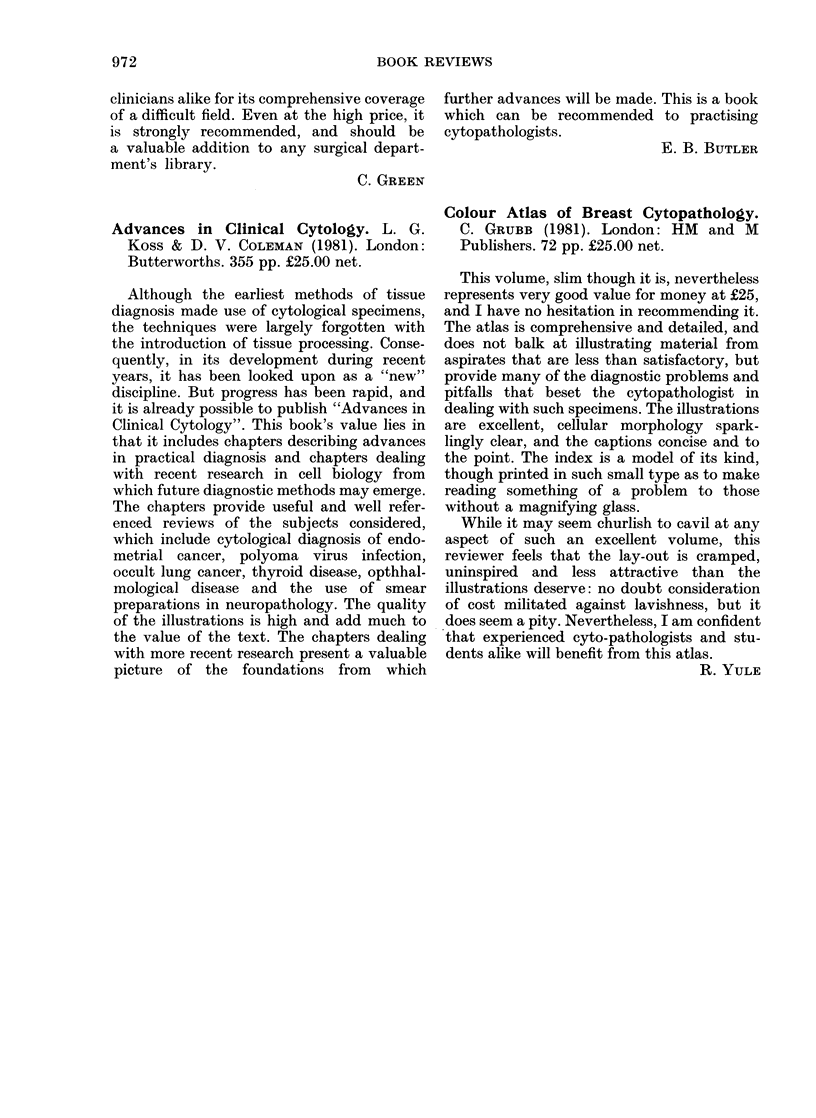# Advances in Clinical Cytology

**Published:** 1982-06

**Authors:** E. B. Butler


**Advances in Clinical Cytology.** L. G. Koss & D. V. Coleman (1981). London: Butterworths. 355 pp. £25.00 net.

Although the earliest methods of tissue diagnosis made use of cytological specimens, the techniques were largely forgotten with the introduction of tissue processing. Consequently, in its development during recent years, it has been looked upon as a "new" discipline. But progress has been rapid, and it is already possible to publish "Advances in Clinical Cytology". This book's value lies in that it includes chapters describing advances in practical diagnosis and chapters dealing with recent research in cell biology from which future diagnostic methods may emerge. The chapters provide useful and well referenced reviews of the subjects considered, which include cytological diagnosis of endometrial cancer, polyoma virus infection, occult lung cancer, thyroid disease, opthhalmological disease and the use of smear preparations in neuropathology. The quality of the illustrations is high and add much to the value of the text. The chapters dealing with more recent research present a valuable picture of the foundations from which further advances will be made. This is a book which can be recommended to practising cytopathologists.

E. B. Butler